# Persistence of Severe Acute Respiratory Syndrome Coronavirus 2 (SARS-CoV-2) Virus and Viral RNA in Relation to Surface Type and Contamination Concentration

**DOI:** 10.1128/AEM.00526-21

**Published:** 2021-06-25

**Authors:** Susan Paton, Antony Spencer, Isobel Garratt, Katy-Anne Thompson, Ikshitaa Dinesh, Paz Aranega-Bou, David Stevenson, Simon Clark, Jake Dunning, Allan Bennett, Thomas Pottage

**Affiliations:** a Public Health England, National Infection Service, Porton Down, Wiltshire, United Kingdom; b Emerging Infections and Zoonoses, National Infection Service, Public Health England, London, United Kingdom; c NIHR Health Protection Research Unit in Emerging and Zoonotic Infections, University of Oxford, Oxford, United Kingdom; University of Queensland

**Keywords:** SARS-CoV-2, environmental persistence, COVID-19, transmission, surface sampling, environmental sampling

## Abstract

The transmission of SARS-CoV-2 is likely to occur through a number of routes, including contact with contaminated surfaces. Many studies have used reverse transcription-PCR (RT-PCR) analysis to detect SARS-CoV-2 RNA on surfaces, but seldom has viable virus been detected. This paper investigates the viability over time of SARS-CoV-2 dried onto a range of materials and compares viability of the virus to RNA copies recovered and whether virus viability is concentration dependent. Viable virus persisted for the longest time on surgical mask material and stainless steel, with a 99.9% reduction in viability by 122 and 114 h, respectively. Viability of SARS-CoV-2 reduced the fastest on a polyester shirt, with a 99.9% reduction within 2.5 h. Viability on the bank note was reduced second fastest, with 99.9% reduction in 75 h. RNA on all surfaces exhibited a 1-log reduction in genome copy number recovery over 21 days. The findings show that SARS-CoV-2 is most stable on nonporous hydrophobic surfaces. RNA is highly stable when dried on surfaces, with only 1-log reduction in recovery over 3 weeks. In comparison, SARS-CoV-2 viability reduced more rapidly, but this loss in viability was found to be independent of starting concentration. Expected levels of SARS-CoV-2 viable environmental surface contamination would lead to undetectable levels within 2 days. Therefore, when RNA is detected on surfaces, it does not directly indicate the presence of viable virus, even at low cycle threshold values.

**IMPORTANCE** This study shows the impact of material type on the viability of SARS-CoV-2 on surfaces. It demonstrates that the decay rate of viable SARS-CoV-2 is independent of starting concentration. However, RNA shows high stability on surfaces over extended periods. This has implications for interpretation of surface sampling results using RT-PCR to determine the possibility of viable virus from a surface, where RT-PCR is not an appropriate technique to determine viable virus. Unless sampled immediately after contamination, it is difficult to align RNA copy numbers to quantity of viable virus on a surface.

## INTRODUCTION

Severe acute respiratory syndrome coronavirus 2 (SARS-CoV-2) causing coronavirus disease 2019 (COVID-19) has spread globally, and many countries are experiencing ongoing local transmission despite various levels of control efforts. SARS-CoV-2 is primarily transmitted via respiratory droplets from an infected host (1). Studies have confirmed aerosol viral transmission (2–4), with SARS-CoV-2 being shown to remain viable in aerosols for between 90 min and 3 h in laboratory studies (5, 6). Infections from direct person-to-person transmission have been confirmed as well as indirect transmission through close contacts after tracing of case clusters (7, 8). It is suspected that contaminated surfaces or fomites also have a role in transmission. Studies detailing SARS-CoV-2 viability on surfaces have contributed to this (6).

SARS-CoV-2 RNA has been detected on environmental surfaces, potentially indicating the presence of viable virus (9, 10). Current environmental sampling of surfaces using swabs primarily uses reverse transcription-PCR (RT-PCR) to detect viral genome in samples. Few studies have been able to isolate viable virus from environmental surface sampling, even where RT-PCR indicates a high level of SARS-CoV-2 RNA is present (11). Recent manuscripts have identified survivability ranges for SARS-CoV-2 on surfaces in the laboratory, with only one demonstrating the relationship between viable recovered virus and RNA on the surface (12, 13). As the risk of infection from virus-contaminated surfaces is difficult to predict (14), further investigation is required to enhance understanding of the survivability of SARS-CoV-2 on surfaces.

The three aims of this study were (i) to measure the persistence of viable SARS-CoV-2 virus on common personal protective equipment (PPE) materials (both hospital-grade and reusable fabrics), high-touch surface materials, and commonly worn fabrics; (ii) to investigate the relationship between recoverable viable virus from these surfaces and the levels of SARS-CoV-2 RNA in the same sample; and (iii) to determine the relationship between inactivation rate and initial viral titer load on surfaces.

## RESULTS

**FIG 1:**
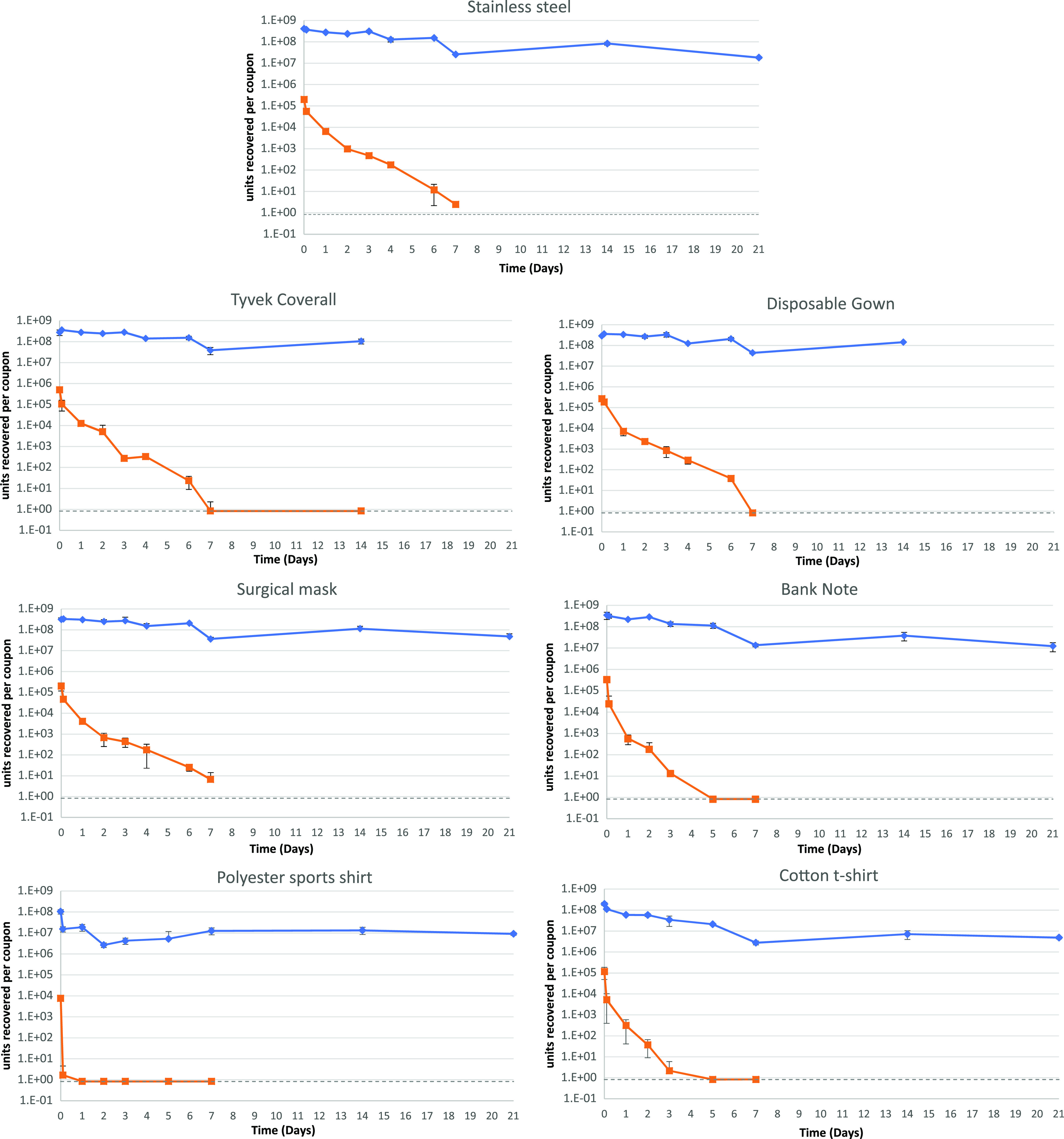
Mean quantities of viable virus recovered (PFU/coupon, orange) and viral RNA detected (genome copy number/coupon, blue) for 7 materials assessed. Error bars represent the standard deviations from three replicates. The gray dashed line represents the limit of detection of the plaque assay for the combined assays from the triplicate coupons (0.8 PFU/ml). For Tyvek coveralls and disposable gowns, 21-day coupons were not processed.

SARS-CoV-2 viability decreased on all materials during the 2.5-h drying period, on average by 1.01 log_10_, with standard deviations across materials of 1.06 log_10_ (range, −0.18 log_10_ for disposable gown to −3.66 log_10_ for polyester sports shirt) from a high starting inoculum of approximately 4 × 10^5^ PFU per material (standard deviation, 2 × 10^5^ PFU). Viable virus could be recovered from the surgical mask and stainless steel coupons for the longest periods of time (log_10_ reductions of 4.91 and 4.99, respectively, over 7 days). The recovery time for cotton t-shirt and polyester sports shirt materials was shorter (log_10_ reductions of 5.15 over 5 days and 3.9 within 1 day). RNA copy number was recovered at higher concentrations in all samples compared to the levels of viable virus, decreasing by ∼1.5 log_10_ (nonporous, hydrophobic) and ∼1 log_10_ (porous, hydrophilic) over the initial 7 days and then stabilized at around 10^7.5^ copies per coupon from day 7. The ratio of viable virus recovered ranged from 10^3^ to 10^8^ times less than the viral RNA assayed from start to finish of the study period (Fig. 1).

Linear regression analysis was completed using the recoverable virus data to calculate time for percent reduction (Table 1). Regressions were calculated from *t* = 2.5 h onwards. Calculated decay rate is fastest on bank notes, with a 99.9% reduction in recovery within 75 h. The longest survival of virus is observed on surgical mask material, where 122 h is required for a reduction of 99.9%. For the polyester sports shirt, a >3-log_10_ reduction was detected during the initial 2.5-h time point (Table 1).

**TABLE 1:** Materials used in this study, their properties, and the time for percent reduction values for the multisurface study, quantified by plaque assay^*a*^

Material	Properties	Log change after 2.5 h of drying	*R*^2^ value	Time for % reduction (h)			SD (all TfPR values)
				90%	99%	99.90%	
Surgical mask	H−, P	−0.64	0.955	44.68	83.32	121.96	0.63
Stainless steel	H−, NP	−0.56	0.982	38.02	75.84	113.66	5.26
Tyvek coverall	H−, NP	−0.7	0.962	39.02	72.65	106.27	9.17
Disposable gown	H−, NP	−0.18	0.946	28.65	63.73	98.82	3.69
Cotton t-shirt	H+, P	−1.34	0.904	33.94	58.97	84.00	9.76
Bank note	H−, NP	−1.13	0.955	27.97	51.42	74.86	4.65
Polyester sports shirt	H+, P	−3.66	NA	<2.5			NA

a Material properties: hydrophobic (H−), hydrophilic (H+), porous (P), and nonporous (NP). TfPR, time for percentage reduction of values for the multisurface study. The final column provides standard deviations, applicable to all 3 values. Data are sorted in descending order by time to 99.9% survival rates. NA, not applicable.

**FIG 2:**
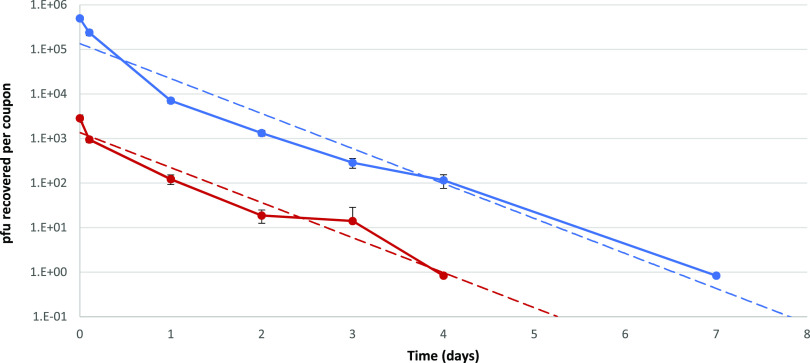
Virus viability results from loading of high (4 × 10^5^ PFU added, blue) and low (4 × 10^3^ PFU added, red) SARS-CoV-2 inoculum onto stainless steel coupons (*n* = 3). Dashed lines show linear regression based on recovery over time.

The results from the comparative study involving two viral titers revealed an initial rapid decrease in the recovery of viable virus from the surface during the drying period, with the inactivation rate decreasing after drying. Virus was recovered after 4 days for the low inoculum and up to 7 days for the high inoculum (Fig. 2). Parallel survival rates of high and low inocula demonstrate that the decay rate of SARS-CoV-2 is independent of concentration when applied to a stainless steel surface; linear regression of high and low inocula over time showed no significant difference between slopes (*P* = 0.29, degrees of freedom = 9).

## DISCUSSION

Contact with SARS-CoV-2-contaminated surfaces is thought to be a route of transmission in the current pandemic (8, 15). Surfaces can be contaminated by virus-containing droplets generated from an infected individual or contact with contaminated hands, with potential onwards transmission via direct surface contact (16). With contamination events likely to occur in a range of materials, this study investigated the survival of SARS-CoV-2 UK isolate England 02/2020 (EPI_ISL_407073) and associated viral RNA on a range of surfaces that are at risk from droplet and touch contamination.

Existing studies examining time-based viability of SARS-CoV-2 on different surfaces have focused on a single virus titer (6, 12, 13, 17). Our work investigated the persistence of high titers of SARS-CoV-2 on various surfaces and at two different titers on stainless steel coupons with identical conditions.

When inoculated onto stainless steel, a 5-log reduction in viability of the UK SARS-CoV-2 isolate England 02/2020 was observed over a 7-day period (Fig. 1). Riddell et al. showed a similar log_10_ reduction at 28 days and Kasloff et al. at 14 days using the Australian isolate Betacoronavirus/Australia/SA01/2020 and Canadian isolate hCoV-19/Canada/ON-VIDO-01/2020, respectively (12, 17). In both studies, the viral propagate included additives such as serum and mucin to mimic bodily secretions (12, 17). In the current study, the virus stock suspension was centrifuged to remove the majority of the cell debris but left salts and proteins from the growth media used to propagate the virus. The initial starting inoculum concentration (between 10^5^ and 10^6^ PFU per surface) and environmental conditions of temperature and relative humidity (RH) are similar for the two previous studies and this study, demonstrating it is likely that the differences observed in survival results primarily arise from a protective effect afforded by added serum/mucin and/or the different isolates used. A difference between the current study and the previous studies was the exposure of the coupons. During Kasloff et al.’s study, the coupons were placed in a vented box and stored within a closed cabinet once dried. In Riddell et al.’s study, the coupons, once dry, were placed in a climate chamber in the dark. The current study held the coupons in a flexible film isolator (FFI) for the exposure period (12, 17). There may have been a detrimental effect on the viability of the virus from the airflow within the FFI compared to the viruses present in the previous studies, but since the virus was dried already on the surface, the desiccation effects would be minimal. The current study took place in a containment level 3 laboratory without external windows; therefore, only low levels of UV light, generated from the laboratory’s compact fluorescent lights, would contact the coupons. This UV level produced would further be reduced due to the distance of the coupons away from the lights. The light must pass through the FFI layers and the indirect line of sight due to air-handling equipment for the FFI. While potentially artificially high, the starting inoculum in this study provides the ability to determine the inactivation characteristics of the virus on the different materials, which a lower starting inoculum may not achieve.

Currently there are no published studies investigating interisolate differences in environmental surface stability of SARS-CoV-2. A study investigating the stability of SARS-CoV-1 (AY274119.3) and SARS-CoV-2 (nCoV-WA1-2020) by van Doremalen et al. showed that both were similar under their experimental test conditions (6). Chin et al. have reported findings similar to those of the work presented in our study using a comparable starting inoculum, also without additional protein (13). Their results showed that infectious SARS-CoV-2 was recovered from a banknote and stainless steel on days 4 and 7, respectively, compared to our results of recoverable virus on day 5 from the banknote and recovery on day 7 from stainless steel. With little evidence of difference in environmental stability between isolates of SARS-CoV-2, the addition of bovine serum albumin and mucin to the inoculating suspensions indicates that additional protein provided a protective effect to the virus during and after drying onto the surfaces (12, 13, 17).

Droplets on nonporous or hydrophobic surfaces dry in a beaded shape, giving a high volume-to-surface area ratio. In such an environment, these droplets can produce a core-shell structure (18), which can concentrate the virus particles, salts, and organic material into smaller clumps (19). Such clumping is seen often in nature due to association with cellular matter or protein (20, 21). These closely associated virus particles are protected from environmental pressures such as desiccation, UV, and heat, which cause inactivation (20). This may also protect the virus from the effects of relative humidity. Previous studies have shown that RH has affected the persistence of SARS-CoV-2 on stainless steel, where at 24°C half-life of SARS-CoV-2 decreased with increasing RH, from 15.3 h at 20% RH to 8.3 h at 80% RH (22). However, on porous or hydrophilic surfaces, the droplets are absorbed into the material across a larger surface area, which will lead to less clumping and to the presentation of individual viral particles; this may confer less protection from the drying effects of the environment, leading to a reduction in the viability of the virus.

While other studies have designated materials porous and nonporous, this may be an oversimplification of the surfaces studied (12, 17). The surface of a surgical mask is porous but is made up of overlapping hydrophobic fibers; similarly, Tyvek material is produced with nonwoven fibers of high-density hydrophobic polyethylene but presents microscopic pores on the surface. In the context of our study, relatively small amounts of liquid are being added to these surfaces. Thus, these small droplets of liquid cannot penetrate into the materials, as their hydrophobicity ensures the droplets of liquids remain on the surface of the material during the drying process, making the surfaces behave more like a nonporous one. Our results show that the porous but hydrophobic surfaces of the surgical mask, and Tyvek coverall produce decay rates similar to those of the nonporous hydrophobic surfaces of stainless steel and disposable gown with a 5-log_10_ reduction in recovered infectivity over 7 days. Viable SARS-CoV-2 was recovered from these surface materials over longer periods of time compared to the truly porous and hydrophilic surfaces tested, cotton and woven polyester. An exception was the hydrophobic polymer bank note, from which viable virus was recovered at the limit of detection for days 5 and 7 of the study. This is different from the study of Riddell et al., where the recovery from the Australian bank note was similar to that of the other nonporous surfaces tested (17). At present, it is not clear why this surface had decreased viability compared to the other nonporous hydrophilic surface used in this study, although there may be antiviral properties from some of the dyes used in the bank note.

Following a 4.73-log_10_ decrease, infectious virus was recovered from cotton material up to 3 days after inoculation, matching previous studies that reported more rapid inactivation of virus particles on cotton surfaces than others (12, 17). These results may be attributed to two factors unrelated to any potential antiviral activity of the material: retention of virus within the cotton fiber matrix and losses during the inoculum application due to wicking. Due to the cotton’s hydrophilic, woven nature, the liquid inoculum rapidly absorbs and penetrates into the fibers, which, when dried, might cause interactive forces, limiting the release of virus particles, which is shown by a greater than 1-log_10_ reduction in recovery of viable virus after the drying period. This decrease in detection of viable virus may be attributable to inefficient recovery from this specific type of material rather than increased inactivation. This result indicates that viral particles remain in cotton fibers after contamination, posing a forward transmission risk, but they will likely not be released from the substrate to cause infection. To counteract the material’s inherent absorbent nature, during the inoculation and drying steps we suspended the cotton in strips across an open box. While this exposed the virus-inoculated coupon to the environmental conditions on both sides of the coupon, it reduced any potential losses of the virus due to wicking on to container surface from the coupon, as seen in a previous study (12).

Although it is hydrophobic, when polyester (produced from polyethylene terephthalate) was spun into fine fibers, aligned in the same orientation, and woven into fabric, it behaved like the other woven fabric tested, cotton. It is possible that the aligned polyester fibers, which are close together but not fused, causes capillary action to draw the liquid into the interstitial spaces between the fibers and trap the virus particles. Virus that was inoculated onto a polyester sports shirt was rapidly inactivated to unrecoverable levels in 1 day; there may also be interaction between the chemicals used to process/color the fabric and the virus (23).

SARS-CoV-2 RNA has been detected on surfaces in different environments, but there have been few reports of viable virus recovery from these surfaces. The use of RT-PCR to determine the presence of SARS-CoV-2 on surfaces has advantages, i.e., increased sensitivity (RT-PCR can detect small amounts of target RNA) and rapid high throughput of samples compared to culture-based methods. The limitation of the use of RT-PCR in such studies is its inability to distinguish between viable and nonviable virus, whereas a positive result from a culture-based method would indicate that an infectious particle was present on the surface at the time of sampling and a there was potential for transmission. Pretreatment of swabs or samples with agents such as propidium monoazide may help reduce amplification of nucleic acid from damaged or degraded viral particles; however, there are limited data available validating their utility for SARS-CoV-2 (24), and assays measuring presence of infectious virus must be prioritized until robust molecular techniques have been developed.

The detection of SARS-CoV-2 RNA from surface samples is used to indicate that virus (viable or nonviable) was present on that surface at some point previously. Lower cycle threshold (*C_T_*) values from the RT-PCR assay indicate that more copy numbers of target RNA are present in that sample. Our study determined that a *C_T_* value of 18 equates to approximately 5 × 10^8^ copy numbers of the RNA target. The initial recovery of infectious virus from the materials (Fig. 1), excluding the polyester sports shirt, is approximately 3.1 log_10_ (standard deviation [SD], 0.13 log_10_) lower than the copy number, showing that there is a large amount of RNA exogenous to infection-competent viral particles in the inoculum. This difference between infectious virus and RNA copy number was also reported by Kasloff et al. (12). The ratio between copy number and viable virus changes with time, with recoverable infectious virus rates reducing more rapidly than copy numbers recovered in the same sample over time. Therefore, the RNA is more persistent in the environment than the infectious virus. It has been shown that SARS-CoV-2 RNA was detected on cruise ship surfaces 17 days after cabins had been vacated (25).

Thus, it is not possible to draw conclusions on the viability of surface contamination from genome copy number of RNA detected after the initial contaminating event. In addition, the comparative persistence of RNA on the surfaces compared to infectivity makes it difficult to relate copy number to the date when the contamination may have occurred. Although our laboratory-based study used a concentration of infectious virus that may not reflect the contamination load present in the environment, we demonstrated recovery of viable virus at high and low concentrations.

Patient nasal and throat swab samples have produced *C_T_* values below 18 (26, 27), even to a *C_T_* value of <12.3 (28), but reported surface samples have produced *C_T_* values above 28 (9, 29–31). Using the results from our study to provide a calculation of the initial viable load on the surface, a *C_T_* of 28 would provide an approximate infectious virus titer of 10^2^ viral particles at the time of immediate surface contamination. Using the recovery results from stainless steel as a representative surface in this study (Fig. 2), infectious virus from an initial recoverable inoculum of 10^2^ viral particles would be unrecoverable within 2 days. This is based on the vortex-mixing recovery method used, and the detection would be thought to reduce further using direct surface sampling methods. Future studies could address this limitation of knowledge where the copy number is determined for much lower concentrations of SARS-CoV-2 on surfaces, which will help to further identify if the persistence of RNA is independent of concentration and address the relationship to viable virus recovery.

### Conclusions.

This study shows that the UK SARS-CoV-2 isolate, England 02/2020, remains viable for longer periods of time on hydrophobic surfaces, up to 7 days, than hydrophilic surfaces, reduced to 3 days, at ambient temperature and relative humidity, indicating that some common surfaces pose an infection risk if contaminated with high concentrations of virus, although viable virus contamination levels of environmental surfaces are likely to be at a low concentration. In contrast, recovery of RNA from the same samples shows little reduction in copy numbers over the same period. The data presented also indicate that the inactivation rate on environmental surfaces is independent of initial loading for SARS-CoV-2 and varies depending on surface type.

## MATERIALS AND METHODS

### Viral isolate.

The SARS-CoV-2 isolate used in this study, England 02/2020 (EPI_ISL_407073) passage 3 (P3), was propagated by the High Containment Microbiology Department at PHE, Porton Down. The virus was isolated from a clinical sample taken during acute-phase illness, using Vero E6 cells (85020206; ECACC). A P2 master bank was produced using Vero E6 cells (NR-596; BEI Resources) and a P3 working bank produced in Vero/hSLAM cells (04091501; ECACC). Cell lines were infected at 95% confluence at a multiplicity of infection (MOI) of 0.0005 to 0.01 and maintained in 1× minimal essential medium, GlutaMAX, 4% heat-treated fetal bovine serum (Gibco), 1× nonessential amino acids (Gibco), 25 mM HEPES buffer (Gibco). Additionally, Vero/hSLAM cells were maintained in the presence of 0.4 mg/ml Geneticin (ThermoFisher Scientific, Gibco). Virus was harvested 3 to 6 days postinfection and supernatant clarified by centrifugation (3,000 rpm, 10 min). Virus was aliquoted and stored at −80°C. The titer of the P3 virus stock was determined to be 2.0 × 10^7^ PFU/ml by plaque assay. All work handling SARS-CoV-2 was performed within a containment level 3 laboratory.

### Preparation of test surfaces.

The surfaces used in this study are representative of nonporous hand-touch sites (stainless steel, 316 grade), a bank note (English polymer £10 note), PPE items used in the hospital and wider environments (multiple layered surgical mask, Tyvek coverall, disposable plastic gown [37310 breathable impervious gown]), and materials representing clothing items, a cotton t-shirt (Fruit of the Loom) and a polyester sports shirt (85% polyester, 15% elastane; Activewear SFP5-M02). Coupons (1 cm by 1 cm) of each material were prepared. Once prepared, nonporous coupons (stainless steel and bank note) were cleaned with Neutracon (NEU5; Scientific Laboratory Supplies, Nottingham, UK) detergent followed by rinsing with 70% isopropyl alcohol (IPA). Additionally, stainless steel was sterilized by autoclaving. The surfaces not suitable for cleaning (disposable plastic gown, cotton t-shirt, polyester sports shirt, and Tyvek coverall) were purchased new and handled aseptically. Materials were subdivided depending on their surface properties (Table 1).

### Inoculation of test surfaces.

Viral aliquots were thawed to room temperature immediately prior to inoculation of the materials. The stock suspension was diluted to 2 × 10^5^ PFU/ml with complete minimal essential medium (cMEM) for low loading or used neat for high loading. Only the high-concentration inoculum was used for the experiments investigating viable virus and RNA recovery on all surfaces, whereas the high and low inocula were applied only to stainless steel for viability with different concentrations. The inoculum was frozen at −80°C and assayed with the generated samples. Coupons were inoculated by the addition of two 10-μl droplets of virus culture within a negative pressure flexible film isolator (FFI) and were left uncovered in plastic petri dishes in the FFI for the duration of the study at a temperature of 21.5°C (±1°C) and an average relative humidity of 45%. Three biological replicate coupons were prepared and inoculated for each time point (0, 2.5, 24, 48, 72, 96, 144, 168, 336, and 504 h) and each material. Triplicate coupons exposed to but not inoculated with the virus acted as negative controls. No viable virus or viral RNA was detected in the negative controls.

### Recovery of SARS-CoV-2 from test surfaces.

Recovery of virus from the coupons at each specified time point was performed by transferring a coupon into 1 ml of cMEM in a 7-ml bijou tube with 4 glass beads (3-mm diameter), followed by vortex mixing for 1 min at maximum speed (Heidolph Multireax vortex). The resulting suspension was transferred to a cryotube for storage at −80°C before analysis. Storage of samples at −80°C was not found to significantly affect viability of the virus or denature the RNA. No significant difference (*P* = 0.345; Mann-Whitney rank sum test) was found between samples processed immediately and those freeze/thawed. Time point zero (*t* = 0) coupons were recovered within 5 min of being inoculated before any drying occurred. The recoveries from the zero time point coupons were used to determine the log_10_ reductions from the subsequent samples.

### Plaque assay.

Coupon recovery liquid was assayed by thawing samples at room temperature and then serially diluted (1 in 10) with MEM (Gibco), 1% l-glutamine (Gibco), 1% nonessential amino acids, and 2.5% 1 M HEPES. A volume of 100 μl of each dilution was pipetted in duplicate (technical replicates) for up to four replicates for neat dilutions (400 μl) onto confluent Vero E6 cells within a 24-well plate (7.9 × 10^4^ cells/cm^2^). After 1 h of incubation (±15 min) at 37°C with plate rocking every 15 to 20 min, 0.5 ml CMC overlay was added to each well, containing 1.5% CMC (3% [wt/vol] carboxymethylcellulose solution in sterile distilled water [Sigma C4888]), 1% antibiotic antimycotic solution (100×) (Sigma-Aldrich), 2× overlay medium (20% 10× MEM [Gibco]), 2% l-glutamine (200 mM) (Gibco), 2% nonessential amino acids (Gibco), 6% sodium bicarbonate solution (Gibco), 8% fetal bovine serum (Sigma-Aldrich), 5% HEPES buffer (Gibco), and 57% distilled water (Versol). After 3 days of incubation at 37°C, cells were fixed with formaldehyde and stained by addition of approximately 250 μl 0.2% crystal violet for 5 min before washing with water. The number of plaques in each well was determined and expressed as plaque-forming units.

### RNA extraction and RT-PCR analysis.

RNA was extracted from aliquots (140 μl) of the coupon recovery liquid using the QIAamp viral RNA minikit (Qiagen Ltd., Manchester, UK). RT-PCR was performed using the VIASURE SARS-CoV-2 real-time PCR detection kit (Viasure; CerTest Biotec, Zaragoza, Spain) by following the methods provided. Quantification was undertaken using the N target with a standard curve generated by serial dilution of an *in vitro* transcript (10).

### Data analysis.

Each time point for each material coupon had 3 biological replicates (individual coupons) and 2 technical replicates (plaque assay performed in duplicate). Calculations for the mean assay counts were determined from these 6 replicates and standard deviations from the biological replicates using Microsoft Excel (Office 365). Time to percent reduction values was calculated using linear regression of PFU/coupon averages in Minitab 18. High and low loading line slopes were analyzed using linear regression analysis with GraphPad Prism software, version 7.
